# Self-Cleaning Ceramic Tiles Produced via Stable Coating of TiO_2_ Nanoparticles

**DOI:** 10.3390/ma11061003

**Published:** 2018-06-13

**Authors:** Amid Shakeri, Darren Yip, Maryam Badv, Sara M. Imani, Mehdi Sanjari, Tohid F. Didar

**Affiliations:** 1 Department of Mechanical Engineering, McMaster University, 1280 Main Street West, Hamilton, ON L8S 4L7, Canada; shakeria@mcmaster.ca (A.S.); yipdp@mcmaster.ca (D.Y.); 2 School of Biomedical Engineering, McMaster University, 1280 Main Street West, Hamilton, ON L8S 4L8, Canada; badvm@mcmaster.ca (M.B.); moetakes@mcmaster.ca (S.M.I.); 3 Nanophyll Inc., 175 Longwood Rd South, Hamilton, ON L8P 0A1, Canada; mehdi@nanophyll.com; 4 Institute for Infectious Disease Research (IIDR), McMaster University, 1280 Main Street West, Hamilton, ON L8S 4L8, Canada

**Keywords:** TiO_2_ nanoparticles, self-cleaning, photocatalyst, heat treatment, APTES treatment

## Abstract

The high photocatalytic power of TiO_2_ nanoparticles has drawn great attention in environmental and medical applications. Coating surfaces with these particles enables us to benefit from self-cleaning properties and decomposition of pollutants. In this paper, two strategies have been introduced to coat ceramic tiles with TiO_2_ nanoparticles, and the self-cleaning effect of the surfaces on degradation of an organic dye under ultraviolent (UV) exposure is investigated. In the first approach, a simple one-step heat treatment method is introduced for coating, and different parameters of the heat treatment process are examined. In the second method, TiO_2_ nanoparticles are first aminosilanized using (3-Aminopropyl)triethoxysilane (APTES) treatment followed by their covalently attachment onto CO_2_ plasma treated ceramic tiles via *N*-(3-Dimethylaminopropyl)-*N*′-ethylcarbodiimide hydrochloride (EDC) and *N*-Hydroxysuccinimide (NHS) chemistry. We monitor TiO_2_ nanoparticle sizes throughout the coating process using dynamic light scattering (DLS) and characterize developed surfaces using X-ray photoelectron spectroscopy (XPS). Moreover, hydrophilicity of the coated surfaces is quantified using a contact angle measurement. It is shown that applying a one-step heat treatment process with the optimum temperature of 200 °C for 5 h results in successful coating of nanoparticles and rapid degradation of dye in a short time. In the second strategy, the APTES treatment creates a stable covalent coating, while the photocatalytic capability of the particles is preserved. The results show that coated ceramic tiles are capable of fully degrading the added dyes under UV exposure in less than 24 h.

## 1. Introduction

TiO_2_ is one of the most well-known photocatalysts, which has been widely used for photodegradation of organic compounds and decomposition of pollutants [1,2,3]. While TiO_2_ with the bulk bandgap of ~3.2 eV (in anatase phase) is transparent to visible light, it could be activated under ultraviolet (UV) light illumination and create photo-generated charge carriers [4,5]. The electrons, which are excited by UV absorption, bring about reduction of oxygen molecules in air and produce superoxide radicals (O_2_•^–^). The superoxide radicals are further reduced to form hydrogen peroxide (H_2_O_2_) and subsequently hydroxyl radicals (OH•) [6,7,8]. On the other hand, oxidation of water molecules by electron holes at the surface of the UV excited TiO_2_ particles can also lead to the formation of hydroxyl radicals and hydrogen peroxide [7,9]. These reactive oxygen species can drive decomposition of organic pollutants and inactivation of micro-organisms, such as *Escherichia coli* cell [10,11,12].

In addition to the photochemical power, TiO_2_ has several other advantages, such as chemical stability, nontoxicity, hydrophilicity and cost, which make it a promising candidate for many industrial and environmental applications, including air purification, water purification, and creation of self-cleaning tiles or glasses used in constructions [9,13,14,15]. From a biomedical perspective, photoexcited TiO_2_ nanoparticles (NPs) have been utilized for cancer cell therapeutics [16,17], self-sterilizing of catheters [18] and antibacterial surfaces, as well as photocatalytic disinfection [19,20].

So far, various physical and chemical approaches have been employed in order to coat TiO_2_ NPs onto different types of surfaces. One of the most commonly used coating methods is the sol-gel deposition technique, which enables a uniform thin TiO_2_ layer on different substrates [21,22,23,24]. Applying organic and inorganic binding agents capable of creating strong adhesion of TiO_2_ is the other widely used strategy to directly coat different substrates with pre-synthesized TiO_2_ particles [25]. Sopyan et al. have added TiO_2_ particles to a paint-like sol composed of fluoropolymer resin and an organotitanium coupling agent. The sol was coated onto a glass substrate and cured at 120 °C [26]. Lin et al. have coated polyvinyl chloride (PVC) polymeric substrate with TiO_2_ particles via dip-coating the substrate into Tetrahydrofuran (THF)-PVC-TiO_2_ suspension [27]. In this method, THF solvent can melt the PVC, providing a strong physical attachment between TiO_2_ particles and the substrate. In another research carried out by Pal et al., TiO_2_ particles were combined with the inorganic binder of potassium silicate, which can create chemical bonds with substrates by a silicification process, and were then applied onto the fired bricks [28].

Silanization of inorganic surfaces with (3-Aminopropyl)triethoxysilane (APTES) is a widely known procedure to functionalize the surfaces with amino terminal groups, which could then be used to anchor different biomolecules (i.e., antibodies and proteins) via creating covalent peptide bonds [29,30,31]. The silanization process creates self-assembled monolayers (SAMs) of desired functional groups onto the surfaces and can be performed via chemical vapor deposition [32,33], liquid phase deposition (LPD) [34] and micro-contact printing [35,36,37]. APTES treatment has also been applied on TiO_2_ particles for various purposes, such as enhancement of proteins immobilization [38], preventing the UV photobleaching [39], and grafting with thermoresponsive polymers which enables TiO_2_ particles to have self-flocculation and temperature-controlled switching photocatalytic properties [40]. However, so far, this method has not been utilized to coat construction materials, such as ceramic tiles with TiO_2_ particles.

Heat treatment is also a determining factor in many TiO_2_ coating techniques. It is possible to precisely control the morphology, distribution, and size of the NPs in TiO_2_ coating via adjusting the heat treatment parameters such as temperature and time [41,42,43]. In addition, heat treatment method has a prominent role in doping TiO_2_ crystal structure with different dopants such as nitrogen and carbon for the sake of photocatalytic activity [44,45].

In this paper, two different strategies were introduced to create a stable covalent coating of TiO_2_ NPs onto ceramic tiles to induce self-cleaning capability. In the first approach, a single heat treatment step was used to make a strong physical bond between the TiO_2_ particles and the ceramic tile. In the second procedure, we used the APTES treatment technique to functionalize TiO_2_ particles, and then the particles were covalently bonded to CO_2_ plasma treated ceramic tiles. In both approaches, we have shown that the photocatalytic power of particles was preserved, the coating was stable, and the surfaces were able to thoroughly degrade the dye that was used as an organic pollutant.

## 2. Materials and Methods

### 2.1. Materials

TiO_2_ particles grade SSP-20 (Sakai Chemical Industry Co., Osaka, Japan) with a fully anatase crystal structure, a pink food dye (A Preema Quality Product, Ingredients: Sodium Chloride and E122 Carmosine), glazed ceramic tiles with the dimension of ~ 3 × 3 cm^2^ (The Home Depot, Hamilton, ON, Canada), triethylamine (Sigma-Aldrich, Oakville, ON, Canada), ammonium hydroxide (Sigma-Aldrich, Oakville, ON, Canada), APTES (Sigma Aldrich, Oakville, ON, Canada), toluene (Sigma-Aldrich, Oakville, ON, Canada), *N*-(3-Dimethylaminopropyl)-*N*′-ethylcarbodiimide hydrochloride (EDC) (Sigma-Aldrich, Oakville, ON, Canada), and *N*-Hydroxysuccinimide (NHS) (Sigma-Aldrich, Oakville, ON,, Canada) were used as received. 

### 2.2. Dye-Degradation Measurements

**Figure 1:**
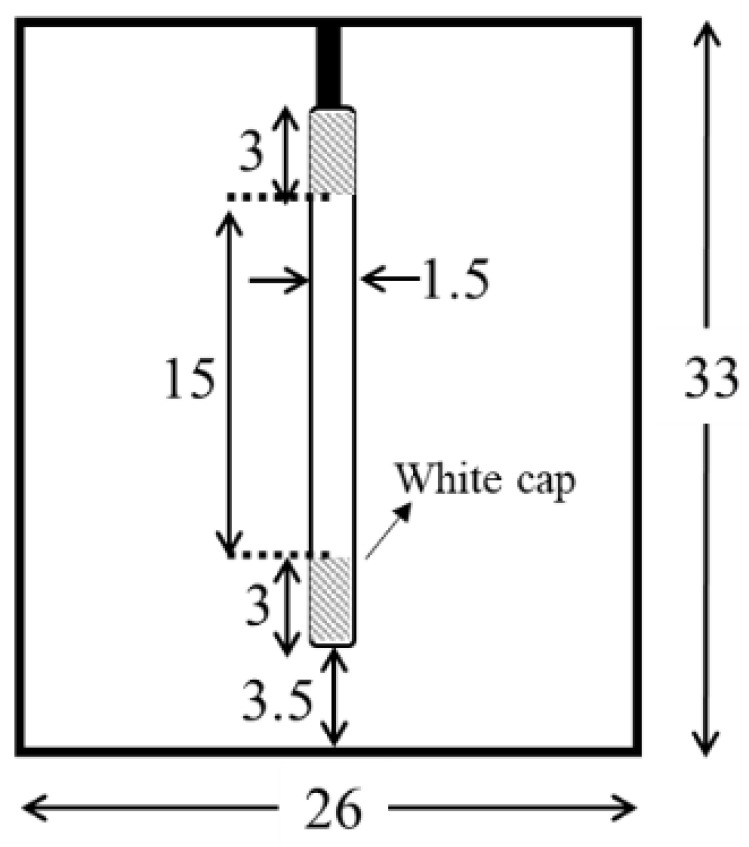
Schematic drawing of the custom-made UV chamber with a UV lamp at the center. All dimensions are in cm.

Dye-degradation experiments were conducted using a custom-made UV chamber (Figure 1). We used a 10-watts Ultraviolet (UV) lamp (Ster-L-Ray, Preheat Germicidal Ultraviolet Lamps, Atlantic Ultraviolet Corporation, Hauppauge, NY, USA) with a total UV output of 2.7 W, a length of 21 cm, and a diameter of ~1.5 cm. The chamber dimensions were 26 × 33 × 25 cm^3^, and the distance from the lamp to the samples was around 3.5 cm. The chamber was covered with an aluminum foil to enhance the reflection.

### 2.3. Dye-Degradation Measurement of TiO_2_ Nanoparticles

The Ultraviolet-Visible (UV-VIS) spectrum of the dye was first measured to determine the optimal wavelength. This was done by using a 1.0 mg/mL concentration of the dye in deionized water and absorbances were read using a plate reader (Infinite M200 Pro, Tecan, Männedorf, Switzerland). The optimal wavelength was determined, based on results obtained using a range of excitation wavelengths. This wavelength was then used to produce a calibration curve to determine the concentration of the dye during the process of cleaning. The calibration graph was produced by measuring the absorbances for dye concentrations of 1, 0.85, 0.70, 0.55, 0.40, 0.25, and 0.1 mg/mL, respectively. Then, TiO_2_ NPs with a concentration of 1 mg/mL in deionized water were mixed with 1 mg/mL of the coloring dye. The suspension was then kept in the UV chamber while stirring to provide consistency with the reaction. A small sample was retrieved from the reaction at 5, 15, 30, 60, 180, and 360 min, and put into a microcentrifuge tube. The tube was then spun at 14,800 rpm for 10 min to pellet the TiO_2_ NPs out of the suspension. Next, 100 μL of the supernatant was gently pipetted from the microcentrifuge tube to the well plate. The well plate was then scanned using the plate reader, and the data obtained was graphed and interpreted.

### 2.4. Coating TiO_2_ Nanoparticles Using Heat Treatment Technique

**Figure 2:**
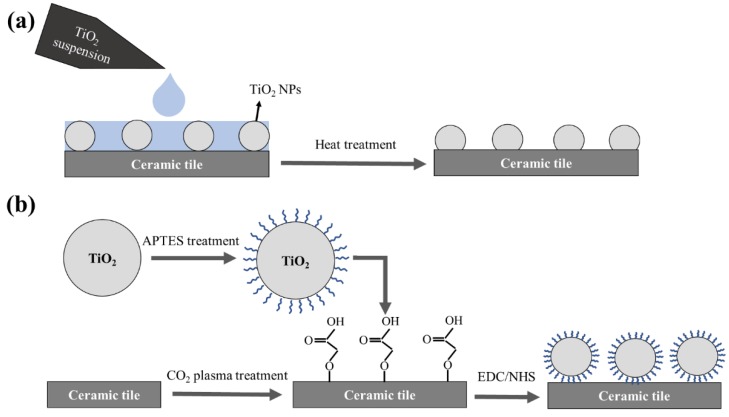
Schematic representation of (**a**) a heat treatment method and (**b**) an APTES treatment method for coating TiO_2_ NPs on ceramic tiles.

Thirty microliter of 1 mg/mL TiO_2_ NPs suspension was added onto the ceramic tiles’ surfaces and spread to cover the whole surface using a simple pipetting system, and then left to dry at room temperature. The surfaces were then placed in a tube furnace or a vacuum oven for lower temperatures. The heat treatments were done in different durations of 2 h and 5 h, and at different temperatures of 100, 200, 400, 500, 700 and 900 °C. The rate of the temperature gradient in all experiments was 5 °C/min. Following heat treatment, the surfaces were rinsed and wiped to remove any unattached particle (Figure 2a). Five microliter of 1 mg/mL of dye was added onto each surface and placed in the UV chamber to evaluate the amount of dye degradation in different time intervals. 

### 2.5. APTES Treatment Technique

TiO_2_ NPs were homogenized with triethylamine (for improving the particle dispersion [46]) and ethanol at 60 °C. Further, ammonium hydroxide, deionized water (DI) water, and APTES (~1%) were added to the suspension and left to stir overnight. [40]. Ammonium hydroxide as a catalyst can increase the rate of APTES hydrolysis reactions [47]. Finally, the suspension was centrifuged at 4500 rpm for 15 min. The supernatant was discarded, and the pellet was mixed with ethanol. The washing step was repeated 3 times and the obtained powder was stored in 50 mL of toluene.

In order to coat ceramic tiles with the APTES treated particles, the cleaned surfaces were CO_2_ plasma treated (Harrick Plasma, Ithaca, NY, USA) to create carboxylic groups onto the surface. EDC-NHS chemistry (a molar ratio of EDC:NHS: ~ 1:1) was used to activate the carboxylic groups on the surfaces. The solution was rinsed off the surface with DI water, and then the APTES treated TiO_2_ suspension was added to the surfaces and left for 2 h. Finally, the surfaces were rinsed with DI water and dried at room temperature (Figure 2b). Five microliter of 1 mg/mL dye was added to the surfaces and left to dry. The surfaces were placed in the UV chamber and imaged at different time intervals. 

### 2.6. Characterization Techniques

High-resolution XPS was used to investigate the amount of carboxylic groups induced by CO_2_ plasma treatment of glass slides for different time durations. XPS measurements were carried out at binding energy between ~280 to ~300 (eV) to capture bond of O–C=O, C=O, C–C, and C–O as counts per second. The size distribution profile of the TiO_2_ NPs in suspension was determined using the dynamic light scattering method (DLS, Beckman Coulter, Indianapolis, IN, USA). In DLS analysis, 10 acquisitions were performed for every experiment, and each acquisition was 15 s long. All TiO_2_ NPs were diluted in DI water with the concentration of 0.01 mg/mL and sonicated for 5 min. For the APTES treated sample, we took 5 μL of the suspension of NPs in toluene and diluted in DI water (10 mL) to achieve the desired concentration in order to exclude the effect of toluene in DLS measurements. Contact angles were measured by an optical contact angle (OCA 35) (Future Digital Scientific Corp., Garden City, NY, USA) using 5 μL droplet of DI water. Fourier-transform infrared spectroscopy (FT-IR) (Bruker, Karlsruhe, Germany) was used to confirm the presence of the TiO_2_ coating on the ceramic tiles’ surfaces after the washing steps as well as the effect of APTES treatment on the TiO_2_ NPs. FT-IR was conducted on different samples and different spots on the surfaces for each experiment. ImageJ was utilized to quantify the photos achieved from the dye-degradation results. For better comparison, the graphs were normalized, so that all the graphs started at the same point. Atomic force microscopy (AFM) (BioScope Catalyst, Bruker, Milton, ON, Canada) was performed to analyze surface topography and roughness within the scan size of 1 × 1 μm^2^. A ScanAsyst mode was utilized in the measurements using a probe with the spring constant of 0.4 N/m.

## 3. Results and Discussion

### 3.1. TiO_2_ NPs Size Measurements

**Figure 3:**
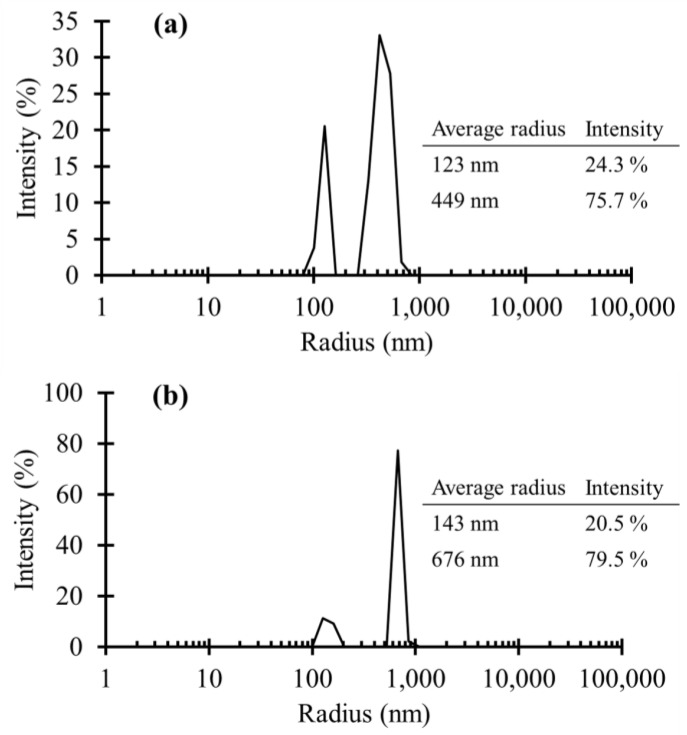
DLS results of the (**a**) untreated TiO_2_ NPs and (**b**) APTES treated TiO_2_ NPs. The vertical axes indicate the particle number percentages. The insect tables show the average radius of the NPs and related particle number intensities.

Figure 3 demonstrates the average particle sizes of untreated TiO_2_ NPs and APTES treated TiO_2_ NPs versus the particles population percentage. As is shown, the average radius of the untreated NPs was mostly distributed in two different sizes of 123 nm (24.3% of particle number) and 449 nm (75.7% of particle number). The existence of the larger particle sizes could be due to the NPs agglomeration even after the sonication. In Figure 3b, there were also two main particle size distributions associated with the APTES treated NPS. At the first peak, the average radius was almost the same as the untreated NPS. However, the number of NPs with that radius decreased, compared to the untreated NPs. Moreover, APTES treatment caused the second distribution to go up in terms of average radius as well as population density of the NPs. Consequently, APTES treatment of the particles caused a slight increase in the particles agglomeration, although this was not significant.

### 3.2. XPS Measurement of the CO_2_ Plasma Treated Ceramic Tiles

The second approach that we employed to covalently bind TiO_2_ NPs to the surface of ceramic tiles was aminosilanization of TiO_2_ NPs and CO_2_ plasma treatment of ceramic tiles. APTES treatment of TiO_2_ NPs formed a monolayer of hydrolyzed and condensed APTES molecules on the surface providing free terminal NH_2_ groups. Schematic presentation of this functionalization process is shown in Figure 2b. On the other hand, CO_2_ plasma treatment of ceramic tiles can produce carboxylic groups (COOH) on the surface, which can subsequently be activated using EDC/NHS chemistry. The functional carboxylic groups can go through a chemical reaction with amino groups of the APTES treated particles and form covalent peptide bonds. Consequently, we would be able to covalently bind the TiO_2_ particles to the ceramic tiles.

**Figure 4:**
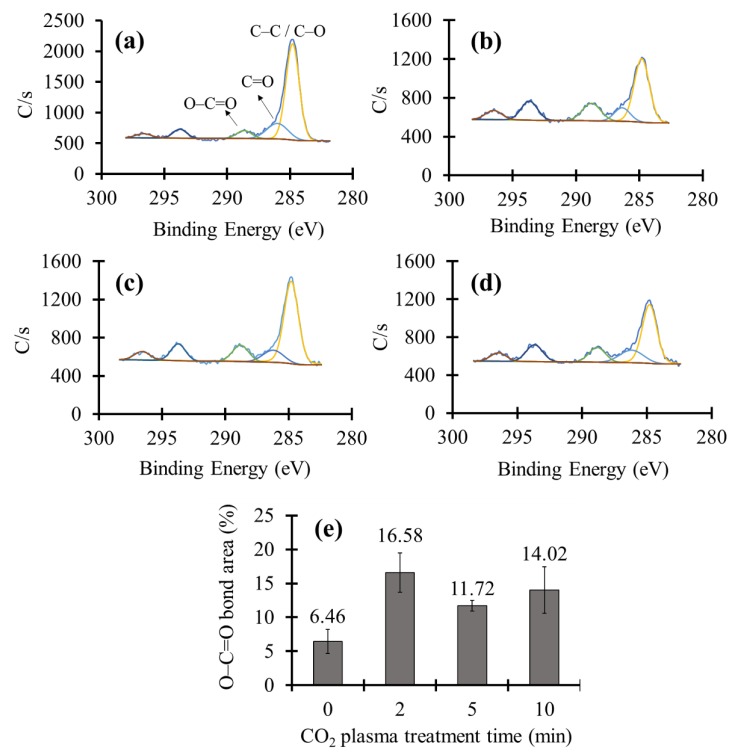
High-resolution XPS plots of CO_2_ plasma treated glass surfaces for (**a**) 0 min; (**b**) 2 min; (**c**) 5 min; and (**d**) 10 min; (**e**) Variation of the amount of O–C=O bonds versus plasma treatment time.

To investigate the carboxylic group formation on the surface, a high-resolution XPS test was performed. Figure 4a depicts the XPS results of an untreated sample. XPS graphs of CO_2_ plasma treated samples for different time periods are shown in Figure 4b–d. The peaks at binding energies around 288.66 (eV), 286.02 (eV), and 284.8 (eV) were assigned to O–C=O, C=O, and C–C as well as C–O, respectively [48]. Figure 4e demonstrates the variation in the amount of O–C=O groups by increasing the CO_2_ plasma treatment time. It could be seen that after 2 min of plasma treatment, the O–C=O band area in XPS patterns has risen from 6.46% to 16.58%, indicating the well formation of carboxylic groups via the CO_2_ plasma treatment. Increasing the plasma treatment time more than 2 min did not significantly change the O–C=O % area.

### 3.3. FT-IR Studies of the TiO_2_ Coated Tiles

**Figure 5:**
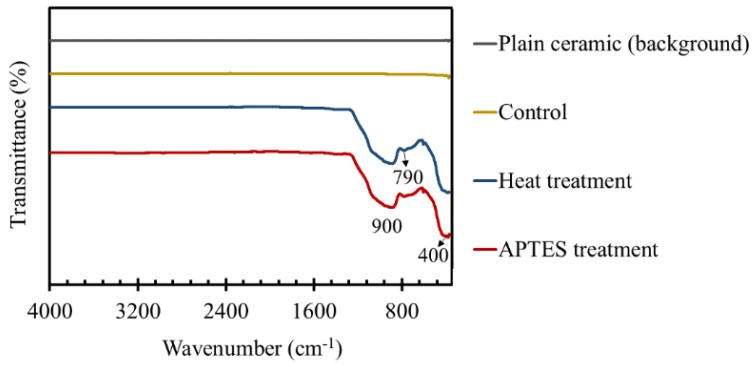
FT-IR spectra of a plain ceramic tile indicated as the background, a control ceramic tile in which untreated particles were applied to the substrate and then the washing steps were conducted, a TiO_2_ coated ceramic tile using the heat treatment method at 200 °C for 5 h, and a TiO_2_ coated ceramic tile using an APTES treatment protocol.

FT-IR spectra of plain ceramic tiles, as well as the tiles coated with TiO_2_ NPs using heat treatment and APTES treatment methods, are plotted in Figure 5. In FT-IR measurements, the surface of a plain ceramic tile was considered to be the background, and all other spectra were normalized based on that. As a control, we applied untreated TiO_2_ NPs on ceramic tiles and then performed the regular washing steps. It can be seen that the obtained spectrum was a flat line similar to the background, indicating a complete removal of the TiO_2_ NPs during the washing process. Nevertheless, the spectra of the TiO_2_ coated ceramic tiles using the heat treatment protocol had a number of peaks in the absorption band between 400 to 1200 cm^−1^, mainly at 400 cm^−1^, 790 cm^−1^, and 900 cm^−1^. This absorption band could be ascribed to the Ti–O stretching and Ti–O–Ti bridging stretching modes [49,50,51,52,53]. Thus, the results demonstrated the presence of TiO_2_ NPs onto the surface of the tiles after performing the washing steps. This is due to the strong adhesion of TiO_2_ NPs to the surface induced via the heat treatment at 200 °C for 5 h (the optimum heat treatment condition based on the dye-degradation results in Figure 10). Figure 5 also depicts the same FT-IR spectra for the APTES treated NPs bonded to the plasma treated ceramic tiles. Moreover, the transmittance percentages of the peaks were almost the same as those of the heat-treated samples, which roughly illustrated the equal mass of TiO_2_ coating. The results confirmed the stable bond formation between TiO_2_ NPs and the substrate that retained the NPs onto the surface during the washing steps.

**Figure 6:**
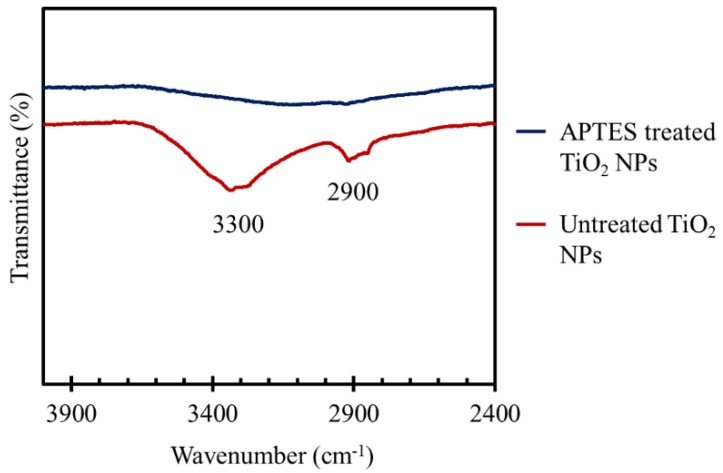
FT-IR spectra of the APTES treated TiO_2_ NPs compared to untreated NPs at the wavenumber between 2400 to 4000 cm^−1^.

In Figure 6, the FT-IR spectra of APTES treated TiO_2_ NPs and untreated NPs were compared at the wavenumber between 2400 to 4000 cm^−1^ to confirm the efficacy of APTES functionalization. There are two main peaks at ~2900 and ~3300 in the transmittance spectrum of untreated TiO_2_ NPs, which indicate the presence of hydroxyl groups on the surface of the NPs. The first peak could be associated with both symmetric and asymmetric CH_2_ stretching vibrations in –CH_2_–OH compound [54], and the second peak was attributed to hydroxyl group symmetric and asymmetric stretching vibrations in Ti–OH compounds [49]. The hydroxyl groups can be formed due to the chemical and physical adsorptions of atmosphere moisture on the surface of NPs [49,55]. As shown in Figure 6, for the APTES treated particles, the peaks related to hydroxyl groups almost disappeared, and a broad peak between 2800 to 3600 cm^−1^ was formed in the spectrum of APTES treated NPs instead. This is because of the hydrolysis and condensation reactions of the silane head groups in APTES molecules and anchoring to the hydroxyl groups of the NPs surfaces to form oxane (Si–O–M) bonds [56]. The broad peak could be caused by OH stretching in silanol (Si–OH) groups (3200–3700 cm^−1^) formed via hydrolysis of APTES, primary amine (3160–3450 cm^−1^), as well as alkane C–H stretching vibrations in –CH_3_ and –CH_2_– (2840–2975 cm^−1^) due to the presence of APTES molecules [54].

### 3.4. Hydrophilicity and Surface Topography of the TiO_2_ Coated Tiles

**Figure 7:**
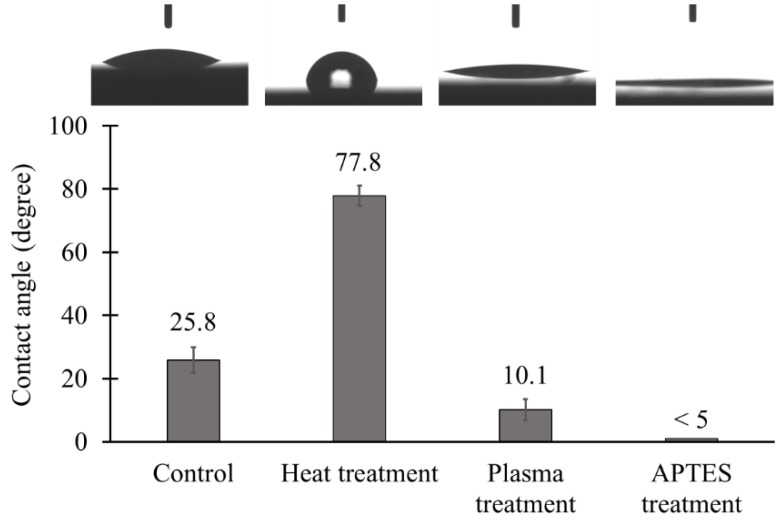
Contact angles of plain ceramic tiles (control), TiO_2_ coated ceramic tiles via the heat treatment method at 200 °C for 5 h, CO_2_ plasma treated ceramic tiles, and TiO_2_ coated ceramic tiles via the APTES treatment method (*n* = 3).

The results of the contact angle measurement are shown in Figure 7. The contact angle of plain ceramic tiles was about 25.8 ± 4.0° indicating the hydrophilic behavior of the surfaces. By performing heat treatment, although surfaces remained hydrophilic, the contact angle rose to 77.8 ± 3.2°. This unexpected increase in contact angle could be due to the surface roughness generated by partial diffusion of TiO_2_ NPs into the surface, which can compensate for the inherent hydrophilicity of TiO_2_ [57]. The APTES treatment method, on the other hand, resulted in more hydrophilic TiO_2_ coated surfaces with the contact angle of less than 5°. A possible explanation for that is CO_2_ plasma treatment step during the APTES treatment protocol, which can greatly increase hydrophilicity as well as the APTES coating on the surface of the NPs [58]. 

**Figure 8:**
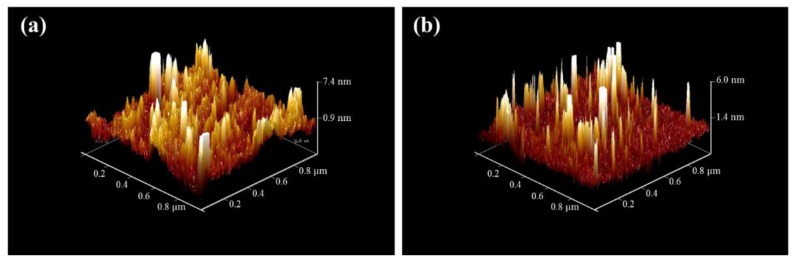
3D AFM images of ceramic tile surfaces coated with TiO_2_ NPs using (**a**) heat treatment at 200 °C for 5 h and (**b**) the APTES treatment method.

AFM images of the TiO_2_ coated ceramic tiles are illustrated in Figure 8. The density of the NPs in the heat-treated sample (Figure 8a) was higher, compared to the sample coated with the APTES treatment protocol (Figure 8b). Furthermore, the root-mean-square (RMS) surface roughness of the ceramic tile increased from 0.676 nm for a plain ceramic to 1.26 nm for TiO_2_ coated ceramic using the APTES treatment method. The RMS parameter of the TiO_2_ coated ceramic tiles with the heat treatment process was 1.82 nm, which was more than that for tiles with the APTES treatment protocol due to the higher number of NPs on the surface.

### 3.5. Dye Degradation of TiO_2_ NPs in Suspension

**Figure 9:**
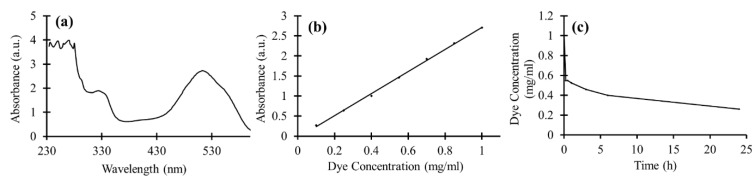
(**a**) UV–VIS spectra of 1 mg/mL concentration of the dye; (**b**) Absorbance curve as a function of dye concentration at the wavelength of 512 nm; (**c**) Dye concentration mixed with TiO_2_ NPs suspension as a function of UV exposure time.

In order to achieve the proper wavelength for reading the absorbances of the dye with a plate reader, the full absorbance spectra of 1 mg/mL concentration of the dye diluted in deionized water was measured. As shown in Figure 9a, the sharp peak at the wavelength of 512 nm, which can be easily distinguished, was used as a reference wavelength for the next absorbance measurements. The calibration curve for dye absorbance as a function of concentration at the wavelength of 512 nm is illustrated in Figure 9b. The obtained scatter diagram has a linear trend line. To measure the photocatalytic power of TiO_2_ NPs, the particles were added to the 1 mg/mL dye solution and were kept in the UV light, under a constant stirring condition. The amount of dye-degradation induced by TiO_2_ photocatalyst could be calculated based on Figure 9b. The dye concentration results versus UV exposure time are shown in Figure 9c. As is shown, a considerable reduction in the dye concentration was achieved after 15 min where almost half of the dye was degraded, and the concentration reached 0.55 mg/mL. Afterwards, the rate of dye degradation became slower and after 1 day of UV irradiation, the dye concentration decreased down to 0.26 mg/mL. Thus, the TiO_2_ nanoparticles possessed a considerable photocatalytic power in suspension. The reason why the expected exponential decay to zero was not observed in our sample could be attributed to the absorption of the partial degradation products on the photocatalyst surface and/or the pH variation of the suspension leading to deterioration in the dye-degradation process.

### 3.6. Dye Degradation on TiO_2_ Coated Tiles Produced Using Heat Treatment

**Figure 10:**
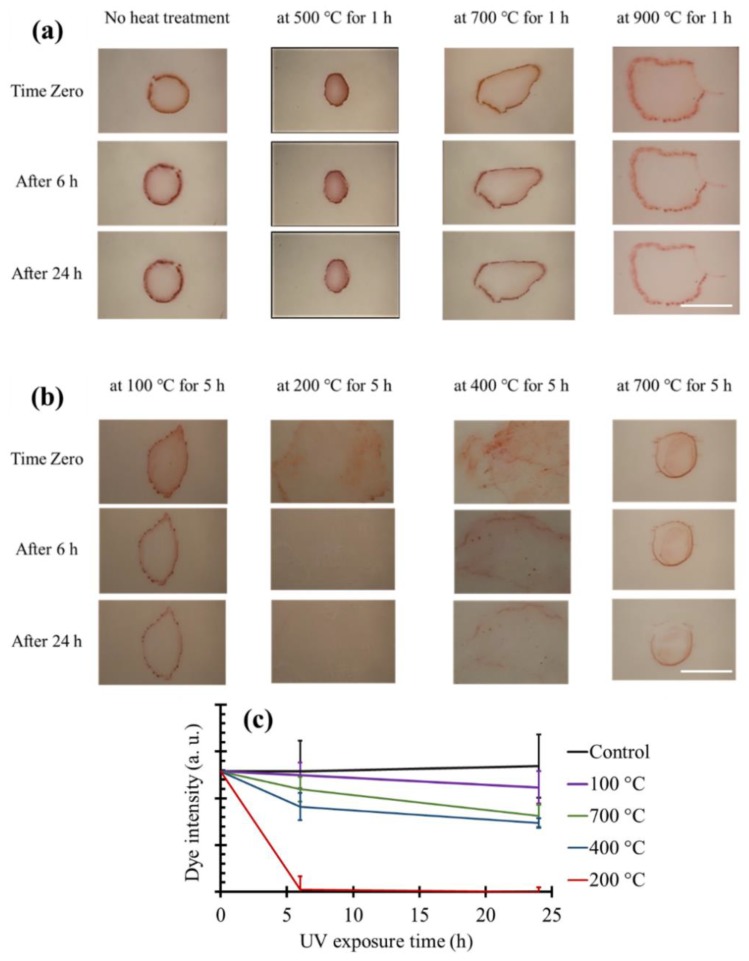
Dye-degradation results of the heat-treated ceramic tiles under UV exposure. Heat treatment was performed at different temperatures for 1 h (**a**) and 5 h (**b**). Scale bars are 1 cm. (**c**) Quantification of the dye degradation on the heat-treated samples for 5 h at different temperatures and the control sample without any heat treatment.

Figure 10 demonstrates the dye-degradation properties of ceramic tiles coated with TiO_2_ nanoparticles using the heat treatment approach at different temperatures and incubation time (1 and 5 h). Control ceramic tiles were incubated with a suspension of TiO_2_ NPs with the same incubation time, but no heat treatment was involved (labeled by “no heat treatment” in Figure 10). Following the washing step, all TiO_2_ NPs were detached from the surface of control tiles and therefore, no dye degradation was observed. Moreover, when the samples were heat treated for 1 h (Figure 10a), no dye-degradation properties were recorded at any heat treatment temperatures. One major problem with high temperatures of 700 °C and 900 °C is that since the temperatures were above the phase transition temperature of TiO_2_ from anatase to rutile [59,60,61,62], the formed rutile phase could have deteriorated the photocatalytic activity of the TiO_2_ particles even if the particles were successfully attached to the ceramic tiles. It has been proved that TiO_2_ exhibits a higher photocatalytic activity in anatase phase compared to fully rutile phase, which may be due to larger band gap in anatase compared to rutile TiO_2_ [4]. Thus, the optimal temperature for heat treatment should not change the structure of TiO_2_. According to the composite phase diagram of TiO_2_, the anatase-rutile phase transition takes place at around 600 °C at atmospheric pressure [59].

Therefore, we increased the heat treatment duration from 1 h to 5 h to investigate the effect of heat treatment time on the coating process, specially the possibility of successful and functional coating at lower temperatures. Figure 10b exhibits the dye-degradation trend for the ceramic tile samples, which were heat treated at varying temperatures for 5 h. In Figure 10c, the results were also quantified for better comparison. The samples that were heat treated at 400 °C degraded the dye at a lower rate than heat treatment at 200 °C, although both temperatures were below the anatase-rutile phase transition temperature. This can be caused by slightly more agglomeration of the TiO_2_ NPs at 400 °C in comparison to 200 °C, leading to a small increase in the average particle size and a reduction in the overall surface area of the particles, thereby diminishing the photocatalytic power. The other possible explanation could be attributed to the higher diffusion of NPs inside the glossy surface of the ceramic tiles at 400 °C. Therefore, the smaller area of the particles would get in touch with the dye, causing a decrease in the dye-degradation rate. Performing the heat treatment at a more cost-effective temperature of 200 °C caused the dye to be fully degraded after 6 h. Nevertheless, 100 °C heat treatment for 5 h appeared to be not enough for the TiO_2_ particles to be completely attached to the surface of ceramic tiles, thereby resulting in less dye degradation under UV exposure. Therefore, a 5 h heat treatment at 200 °C provided the best dye-degradation properties for the ceramic tiles. 

### 3.7. Dye Degradation on TiO_2_ Coated Tiles Produced Using Aminosilanization

**Figure 11:**
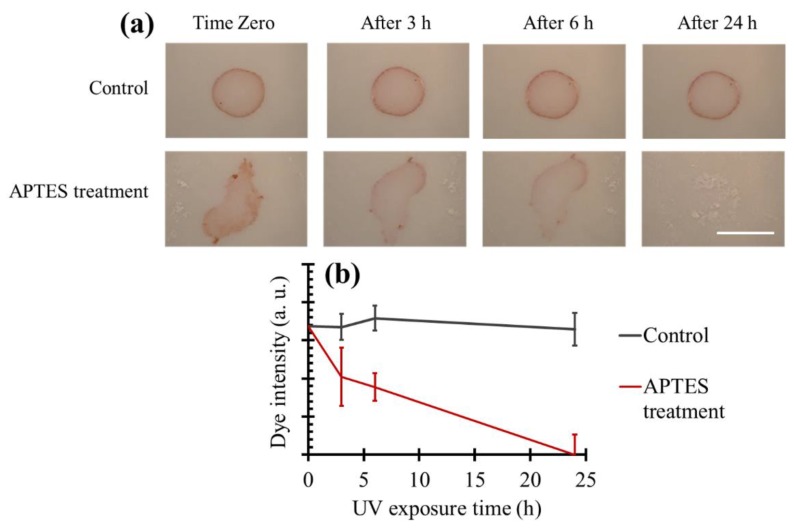
(**a**) Dye-degradation results of the ceramic tiles coated with TiO_2_ particles using the APTES treatment technique. Scale bar is 1 cm. (**b**) Quantification of the dye-degradation images.

Figure 11a illustrates the dye-degradation results of the ceramic tiles coated with the APTES treatment method. Quantifications of the obtained results are plotted in Figure 11b. We applied untreated TiO_2_ particles onto the ceramic tiles as control samples. Most of the particles were removed from the surface during the washing process, and the dye was not degraded after 1 day in control samples. However, when APTES treated particles were used, the dye was almost invisible after 24 h, demonstrating excellent effectivity of the APTES treatment process for coating ceramic tiles with TiO_2_ NPs. In comparison to the heat treatment at 200 °C for 5 h, coating the surfaces via the APTES treatment resulted in a lower rate of dye degradation due to two main reasons: a smaller number of particles on the surface which was confirmed with AFM test, and covering the particles via a silane coupling agent which can reduce the photoactivity of the particles [63]. Nevertheless, using the APTES treatment protocol has two important advantages. First, the method for functionalization using APTES is much simpler and does not require high temperatures compared to the heat treatment. Second, since this technique creates covalent bonds between TiO_2_ NPs and surfaces, the durability of the coating would be higher in the long term.

## 4. Conclusions

The photocatalytic power of TiO_2_ NPs measured inside the suspension showed a proper dye-degradation effect under UV exposure. We introduced two strategies to coat ceramic tiles with the TiO_2_ NPs. Both protocols led to transparent TiO_2_ coating on ceramic tiles without any adverse effect on the tile’s physical appearance. In the heat treatment approach, it was shown that applying a simple one-step heat treatment with the optimum temperature of 200 °C for 5 h resulted in rapid degradation of dye in a short time. In the second strategy, the APTES treatment method was employed to covalently bond the TiO_2_ particles to the CO_2_ treated ceramic tiles, while the photocatalytic power of the particles was preserved. The success of the covalent bonding protocol suggests a possible longer duration of the coating in harsh environments. In future, we will focus on enhancement of the self-cleaning behavior, as well as studying the durability of the coating by performing proper weathering tests.
